# Dynamic evaluation on slope ecological restoration effect based on cosine similarity and markov chain

**DOI:** 10.1038/s41598-023-40770-w

**Published:** 2023-08-19

**Authors:** Wenqiang Chen, Yongai Sun, Kaihe Shi

**Affiliations:** 1 School of Management, Tianjin University of Technology, Tianjin, People’s Republic of China; 2 School of Optometry, Tianjin Vocational Institute, Tianjin, People’s Republic of China

**Keywords:** Ecology, Mathematics and computing

## Abstract

It is important to evaluate the slope ecological restoration effect for diagnosing the slope restoration state in time. Several soft computing methods require experts to determine the index weight, which will affect the rationality of the evaluation results. Moreover, they are all static evaluation methods and cannot reflect the time effect of restoration. Therefore, a dynamic evaluation method has been proposed without determining the index weight based on Cosine Similarity and Markov Chain. Several cases were applied to prove the effectiveness of the proposed method. The results presented that the results of this method are more consistent with the actual situations and can reflect the variability of the restoration effect. Finally, the sensitivity of indexes under different ecological restoration methods was analyzed. The results show that the core link of the restoration method was consistent with the sensitivity result. The proposed method provides a basis for optimizing the restoration methods.

## Introduction

The ecological damage of slope in highway construction, open-pit mine and dam construction has a strong negative impact on the environment, resulting in vegetation loss and soil erosion^1,2^. It is a challenging issue in the field of geotechnical and ecological engineering to effectively implement protection for slopes with instability status to restore damaged vegetation and reduce soil erosion^3^. The ecological restoration of slope mainly refers to the technology of combining living plants with engineering measures to prevent the weathering and peeling of slope, such as vegetation concrete ecological protection technology^4^, tape planting slope technology^5^, CS higher-order granulation technology^6^. It is of great significance to choose and use the appropriate ecological restoration technologies to guide the subsequent slope ecological protection work. However, the research results of slope ecological restoration mainly focus on the selection, design, optimization and mechanism of slope restoration measures, while the overall health status and maintenance of existing ecological restoration projects are rarely studied. In the early stage of ecological restoration, the survival rate and coverage rate of vegetation are greatly affected by climate and seasonal rainfall, which may lead to further deterioration of the ecological environment of the slope. Without timely and accurate health diagnosis and reasonable maintenance, soil and water loss and landslide will be caused, resulting in huge economic losses.

At present, some scholars have studied the health state of slope ecological restoration. However, most of the existing evaluation data are simple and extensive, relying on visual interpretation or qualitative description or rough judgment in the way of expert scoring, and drawing qualitative conclusions such as “excellent”, “good”, “medium”, “poor” and “success or failure”^7^. This evaluation method is greatly affected by individual subjective feelings, and lacks objectivity, accurate, scientific and standardized quantitative standards and evaluation data. Therefore, for the same project, the results obtained by different evaluators are often quite different, and the credibility of the evaluation results is questionable.

Generally, the effect of slope ecological restoration is not only influenced by quantifiable indexes (e.g., soil chemistry, soil physics, species diversity, vegetation coverage, root reinforcement), but also by more qualitative indexes (e.g., soil type, vegetation community, drainage system, landscape coordination, landscape capacity for visitors)^8–11^. This brings great difficulties to the evaluation of ecological restoration effect of slope. Currently, many soft computing methods, such as analytic hierarchy process (AHP) approach^12^, fuzzy mathematical programming method^13,14^, grey clustering analysis method^15,16^, extenics theory^17^, unascertained-set pair^18^, have been widely applied to the evaluation of slope ecological restoration effect, which can take into account quantitative and qualitative indicators comprehensively. However, these soft methods need to determine the weight of indicators through expert personal experiences, which also brings uncertainty to the calculation results. Moreover, these methods cannot simulate the uncertainty factors in the evaluation model, and cannot express the uncertainty of the results more intuitively. In addition, on a time scale, many ecological restoration researchers have reached a consensus on the need to monitor and evaluate changes and improvements in vegetation over time, which is conducive to real-time information in the implementation of vegetation reconstruction projects. However, most of the existing researches are limited to static evaluation, and lack of dynamic evaluation of the change process of slope vegetation.

Cosine Similarity (CS) is a relatively simple clustering algorithm, which is mainly used in the text space index, semantic similarity calculation, fault detection, and so on. CS is also more and more applied in the field of multi-attribute decision making. Long et al.^19^ established a tool selection model based on CS. Liao and Xu^20^ have proposed a CS-based approach to the multi-criterion decision problem of hesitant fuzzy languages and applied the proposed model to the selection of ERP systems. CS is usually defined as the inner product of two non-zero vectors divided by the product of their lengths^21^. If the cosine distance between two vectors is smaller, the similarity between two vectors will be larger. In addition, in multi-attribute decision making, this method does not need to determine the weight of indicators, which minimizes the influence of subjective judgment of experts. At present, there are few researches on the evaluation of slope ecological restoration by CS. The evaluation of the effect of slope ecological restoration is essentially to judge the close degree between the actual state and the ideal state of slope ecological restoration. The higher the proximity degree, the closer the ecological restoration indexes of the slope are to the ideal state, the better the effect is. Therefore, it is feasible to evaluate the ecological restoration effect of slope by CS in this paper. However, CS can only be used for static evaluation. Markov chain (MC) provides a feasible way for dynamic evaluation. MC is a mathematical model that describes dynamic random phenomena, and also an analysis that uses the present state and trend of a variable to predict the future state and trend of the variable, which has a wide range of applications in the fields of economics, sociology and life science. Lu and Chen^22^ proposed a method to predict the dynamic change of urban ecological footprint using MC. Ma and Wang^23^ simulated the future ecological spatial distribution of Wuhan based on MC. Thus, in this research work, we propose a method for dynamically evaluating the effect of ecological restoration of slope without determining weights of indexes based on CS and MC.

The objectives of the study are (1) to establish a dynamic evaluation model of slope ecological restoration effect based on CS and MC; (2) to demonstrate the feasibility of the proposed model and to estimate the effect of slope ecological restoration based on some actual projects; (3) to discuss the sensitivity of evaluation indexes under different slope ecological restoration methods and compare with the actual situation.

## Methodology

### CS evaluation model

CS is the cosine of the angle between two vectors in the vector space as a measure of the difference between two subjects. It can be used in multi-attribute decision analysis to judge the order of pros and cons of each scheme by comparing the cosine of the angle between each scheme and the ideal scheme. Here, a scheme (***Ô***) to be evaluated is assumed. If the ideal scheme is given, the distance between the scheme to be evaluated and the ideal scheme can be determined by the cosine of the angle.

When an evaluation grade standard is given, the index data of the object can be randomly generated coming from the same grade interval based on Monte Carlo simulation (MCS). MCS is an advanced simulation technology, which is used for numerical estimation under the guidance of probability theory and mathematical statistics. This method can scientifically and reasonably solve complex problems with multiple factors and uncertainties. In this way several objects with known evaluation results can be generated by MCS. Then, according to the objects with known evaluation results, the evaluation results of the investigated schemes can be easily calculated by the cosine similarity measures. Herein there is an evaluation grade standard with five evaluation grade and *j* indexes, as shown in Table 1.

**Table 1:** The evaluation grade standard.

Index	Grade				
	I Very good	II Good	III Normal	IV Poor	V Very poor
χ_1_	(χ_10_, χ_11_]	(χ_11_, χ_12_]	(χ_12_, χ_13_]	(χ_13_, χ_14_]	(χ_14_, χ_15_]
χ_2_	(χ_20_, χ_21_]	(χ_21_, χ_22_]	(χ_22_, χ_23_]	(χ_23_, χ_24_]	(χ_24_, χ_25_]
χ_3_	(χ_30_, χ_31_]	(χ_31_, χ_32_]	(χ_32_, χ_33_]	(χ_33_, χ_34_]	(χ_34_, χ_35_]
┆	┆	┆	┆	┆	┆
χ_*j*_	(χ_j0_, χ_j1_]	(χ_j1_, χ_j2_]	(χ_j2_, χ_j3_]	(χ_j3_, χ_j4_]	(χ_j4_, χ_j5_]

Five evaluation objects (***O***^1^,*** O***^2^,*** O***^3^,*** O***^4^, and ***O***^5^) can be dynamically generated based on stochastic simulation, following the uniform distribution (*the number of randomly generated evaluation objects is equal to the number of evaluation grade*)^24^. It is noticed that each index value of the five evaluation objects (***O***^1^,*** O***^2^,*** O***^3^,*** O***^4^, and ***O***^5^) comes from the same grade interval respectively. For example, each index value of ***O***^1^ randomly comes from the first-grade interval, obeying the uniform distribution (*χ*_*j*0_, *χ*_*j*1_], each index value of ***O***^2^ randomly comes from the second-grade interval, obeying the uniform distribution (*χ*_*j*1_, *χ*_*j*2_], and so on, as shown in Table 2. Therefore, the evaluation results of*** O***^1^,*** O***^2^,*** O***^3^,*** O***^4^, and ***O***^5^ are determinable, which corresponds to very good (I), good (II), normal (III), poor (IV), very poor (V), respectively.

**Table 2:** Index data of the randomly generated evaluation objects.

Index	***O***^1^	***O***^2^	***O***^3^	***O***^4^	***O***^5^	function
χ_1_	χ_10_–χ_11_	χ_11_–χ_12_	χ_12_–χ_13_	χ_13_–χ_14_	χ_14_–χ_15_	Uniform
χ_2_	χ_20_–χ_21_	χ_21_–χ_22_	χ_22_–χ_23_	χ_23_–χ_24_	χ_24_–χ_25_	
χ_3_	χ_30_–χ_31_	χ_31_–χ_32_	χ_32_–χ_33_	χ_33_–χ_34_	χ_34_–χ_35_	
┆	┆	┆	┆	┆	┆	
χ_*j*_	χ_*j*0_–χ_*j*1_	χ_*j*1_–χ_*j*2_	χ_*j*2_–χ_*j*3_	χ_*j*3_–χ_*j*4_	χ_*j*4_–χ_*j*5_	
Evaluation result	I	II	III	IV	V	

Cosine distance can be applied to calculate the similarity of the investigated object (***Ô***) with respect to the five evaluation objects (***O***^1^,*** O***^2^,*** O***^3^,*** O***^4^, and ***O***^5^) respectively. Sqrt-cosine similarity measurement can be considered an effective distance measurement in machine learning for high-dimensional applications based on Hellinger distance^25^. Thus, *sim*(***O***^*i*^, ***Ô***), *i* = 1, 2, 3, 4, 5, can be described as:

1 $$sim\left( {{\varvec{O}}^{i} ,\mathop {\varvec{O}}\limits^{ \wedge } } \right) = \frac{{\sum\limits_{j = 1}^{5} {\sqrt {\mathop O\limits^{ \wedge }_{j} \times O_{j}^{i} } } }}{{\sqrt {\sum\limits_{j = 1}^{5} {\left( {\mathop O\limits^{ \wedge }_{j} } \right)} } \times \sqrt {\sum\limits_{j = 1}^{5} {\left( {O_{j}^{i} } \right)} } }},\quad i = 1,2,3,4,5$$

where *sim*(***O***^*i*^, ***Ô***) is the cosine similarity between two objects*** O***^*i*^ and ***Ô***. *Ô*_*j*_ is the *j*th element of the investigated object (***Ô***).

According to the critical similarity value *sim*(***O***^*i*^, ***Ô***)*, If *sim*(***O***^*i*^, ***Ô***) ≥ *sim*(***O***^*i*^, ***Ô***)*, then ***O***^*i*^ and ***Ô*** are the same evaluation result. According to the formula of sqrt-cosine similarity measurement, a proposition can be easily obtained.

#### Proposition

There are three classifications of evaluation objects:*** O***^*i*^, ***O***^*j*^ and ***O***^*k*^. If the classification results of ***O***^*i*^ and ***O***^*j*^ are same, the classification results of*** O***^*i*^ and ***O***^*k*^ are different, then min{*sim*(***O***^*i*^, ***O***^*j*^)} > max{*sim*(***O***^*i*^, ***O***^*k*^)}.

#### Proof

Assume that min{*sim*(***O***^*i*^, ***O***^*j*^)} ≤ max{*sim*(***O***^*i*^, ***O***^*k*^)}.

then, *sim*(***O***^*i*^, ***O***^*j*^) ≥ *sim*(***O***^*i*^, ***O***^*j*^)*, *sim*(***O***^*i*^, ***O***^*j*^)* ≤ min{*sim*(***O***^*i*^, ***O***^*j*^)} ≤ max{*sim*(***O***^*i*^, ***O***^*k*^)}.

Thus, ***O***^*i*^ and ***O***^*k*^ are the same classification, which contradicts the proposition. Therefore, the proposition mentioned above is true.

According to the Proposition, the evaluation result of ***Ô*** can be obtained by calculating max{*sim*(***O***^*i*^, ***Ô***)}, *i* = 1, 2, 3, 4, 5, as follow:

2 $$\left\{ \begin{gathered} Grade\left( {\text{I}} \right),\quad if\;\max \left\{ {sim\left( {{\varvec{O}}^{i} ,\mathop {\varvec{O}}\limits^{ \wedge } } \right)} \right\} = sim\left( {{\varvec{O}}^{1} ,\mathop {\varvec{O}}\limits^{ \wedge } } \right) \hfill \\ Grade\left( {{\text{II}}} \right),\quad if\;\max \left\{ {sim\left( {{\varvec{O}}^{i} ,\mathop {\varvec{O}}\limits^{ \wedge } } \right)} \right\} = sim\left( {{\varvec{O}}^{2} ,\mathop {\varvec{O}}\limits^{ \wedge } } \right) \hfill \\ Grade\left( {{\text{III}}} \right),\quad if\;\max \left\{ {sim\left( {{\varvec{O}}^{i} ,\mathop {\varvec{O}}\limits^{ \wedge } } \right)} \right\} = sim\left( {{\varvec{O}}^{3} ,\mathop {\varvec{O}}\limits^{ \wedge } } \right) \hfill \\ Grade\left( {{\text{IV}}} \right),\quad if\;\max \left\{ {sim\left( {{\varvec{O}}^{i} ,\mathop {\varvec{O}}\limits^{ \wedge } } \right)} \right\} = sim\left( {{\varvec{O}}^{4} ,\mathop {\varvec{O}}\limits^{ \wedge } } \right) \hfill \\ Grade\left( {\text{V}} \right),\quad if\;\max \left\{ {sim\left( {{\varvec{O}}^{i} ,\mathop {\varvec{O}}\limits^{ \wedge } } \right)} \right\} = sim\left( {{\varvec{O}}^{5} ,\mathop {\varvec{O}}\limits^{ \wedge } } \right) \hfill \\ \end{gathered} \right.$$

The uncertainty of five evaluation objects will influence the similarity results, because the indexes values of five evaluation objects are generated using stochastic simulation strategy.

Suppose *Num*_*grade*(*k*)_ (*k* = I, II, III, IV, V) is the random trial result of ***Ô***. For a sequence of *N* tests, *Num*_*grade*(*k*)_ notes the occurrences numbers of *grade* (*k*) (*k* = I, II, III, IV, V). Then the probability of *grade* (*k*) of ***Ô*** in the tests can be calculated as:

3 $$P\left( k \right) = \frac{{Num_{grade\left( k \right)} }}{N},\quad k = {\text{I}},{\text{II}},{\text{III}},{\text{IV}},{\text{V}}$$

Thus, the grade corresponding to the maximum probability is the evaluation result of ***Ô***.

### Dynamic evaluation model of MC

Markov process is a special random motion process. A process of change in *X* of a moving system is called a Markov process if the state of *X*_*r*+1_is only related to the state of *X*_*r*_ and not to the previous state of *X*_*r*_.

4 $$X_{r + 1} = X_{r} \times P$$

where *P* is the transition probability matrix of the process.

5 $$P = \left[ {\begin{array}{*{20}c} {P_{11} } & \cdots & {P_{1n} } \\ \vdots & \ddots & \vdots \\ {P_{n1} } & \cdots & {P_{nn} } \\ \end{array} } \right]$$

Markov process has no aftereffect and stability, and its key lies in the determination of transition probability matrix *P*. *P*_*uv*_ is the transition probability of type *u* to type *v*. *P*_*uv*_ should meet two basic conditions: ① *P*_*uv*_ ∈ [0,1]; ② ∑*P*_*uv*_ = 1.

Since the state of the evaluation result is divided into five levels in part 2.1, the state space is composed of five states in MC model. The transition probability matrix *P* can be expressed as follows:

6 $$P = \left[ {\begin{array}{*{20}c} {P_{11} } & \cdots & {P_{15} } \\ \vdots & \ddots & \vdots \\ {P_{51} } & \cdots & {P_{55} } \\ \end{array} } \right]$$

The evaluation result of ***Ô*** (*P*(I), *P*(II), *P*(III), *P*(IV), *P*(V)) can be used as the initial state in MC model. At present, many researchers determine the transformation probability matrix *P* of MC model based on the multi-year state transformation data. For the absence of multi-year historical data of slope ecological restoration, this paper establishes a constraint model for solving the transformation probability matrix *P* of MC model.

There are two assumptions, as follows:

① The probabilities of transitions between states are not equal;
② The state *X*_1_(0, 0, 0, 0, 1) or *X*_1_(1, 0, 0, 0, 0) will not change after several steps. That is to say, when the evaluation result probability of V or I is 100%, its state will not change with time.

Based on these two assumptions, we establish the objective function and the constraint function.

7 $$\min Q = \sum\limits_{r = 1}^{m} {\sum\limits_{k = 1}^{5} {\left| {\frac{{S_{k} \left( r \right) - \mathop S\limits^{ \wedge }_{k} \left( r \right)}}{{S_{k} \left( r \right)}}} \right|} }$$

8 $$\left\{ {\begin{array}{*{20}c} {\sum\limits_{v = 1}^{n} {P_{uv} = 1,\quad u = 1,{2,} \ldots {,}n} } \\ {P_{uv} \ge 0,\quad u,v = 1,{2,} \ldots {,}n \, } \\ \end{array} } \right.$$

where *S*_*k*_(*r*) is the actual value at state *k* after *r*-step transfer, *Ŝ*_*k*_(*r*) is the estimated value at state *k* after *r*-step transfer.

By solving the above model, the transformation probability matrix *P* of MC model can be obtained. Based on this, it is possible to construct a dynamic evaluation model for MC.

### Calculation procedure

**Figure 1:**
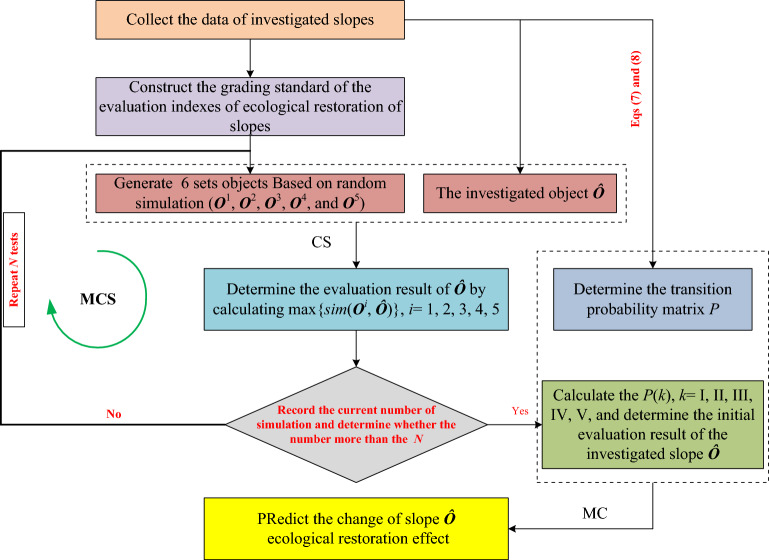
Basic flow chart of the proposed method.

The dynamic evaluation process of slope ecological restoration effect includes two parts, the first part is the initial evaluation using CS, and the second part is the dynamic evaluation using MC. In order to obtain the dynamic evaluation results, the initial evaluation of slope ecological restoration is carried out based on CS firstly. Then, the transition probability matrix was determined based on Eqs. (7) and (8). Finally, according to the initial evaluation results of slope ecological restoration and the transition probability matrix* P*, the dynamic evaluation results were solved by Eq. (4), and the change of slope ecological restoration effect was predicted to realize dynamic evaluation. Based on the above calculation process, we integrated CS and MC to construct a method system for dynamic evaluation of slope ecological restoration. The most critical part of the first part is to randomly generate schemes known evaluation results. Crystal Ball is an easy-to-use simulation software that can run Monte Carlo simulation (MCS) for stochastic simulations, and is associated with Microsoft Excel^®^ spreadsheet program^26^. Therefore, the proposed evaluation method for effect of the slope ecological restoration can easily be carried out by Crystal Ball in an Excel^®^ worksheet. The detailed calculation process is shown in Fig. 1.

## Engineering application

### Background

In Caidian district, Wuhan City, China, there are a lot of mining slopes, and the damaged exposed rock slopes have a significant negative impact on the local natural landscape and ecosystem. From 2006 to 2013, the government forced the closure of all quarries in the local area, and carried out large-scale ecological restoration of damaged mountains, with a total area of 360,000 m^2^ and a total investment of nearly USD 9.8 million.

**Figure 2:**
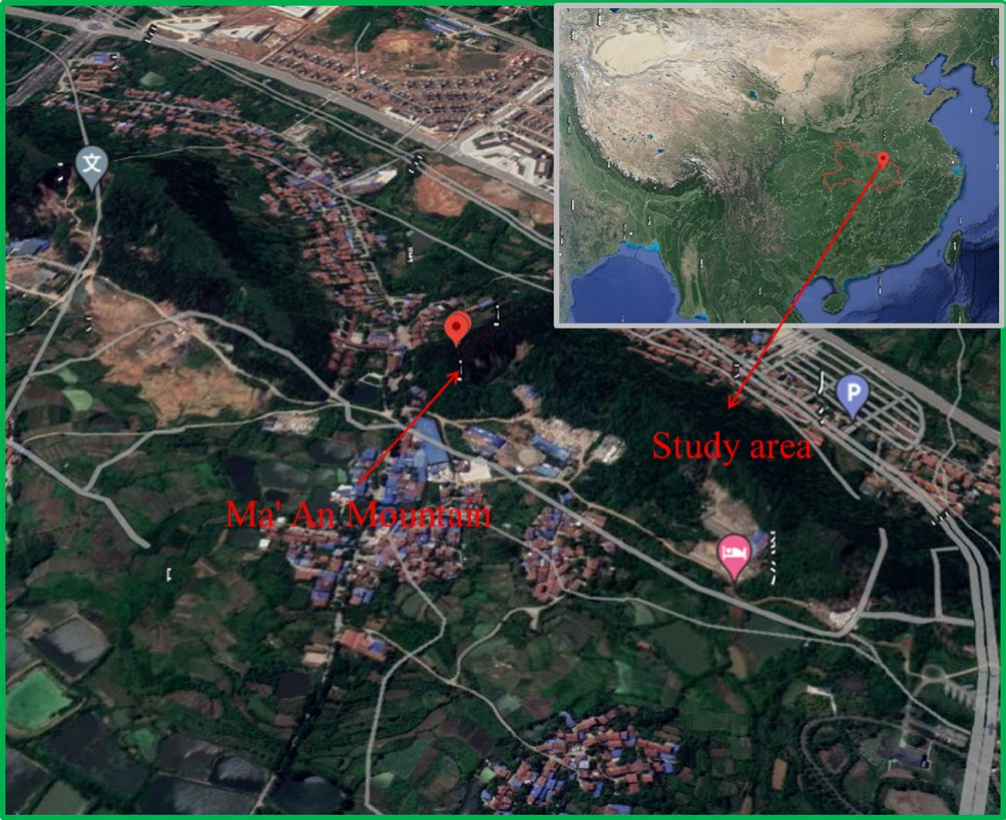
The geographical location of the study region (Google Earth, 2020).

We applied the proposed evaluation model to seven completed slope ecological restoration projects with different restoration methods. Figure 2 shows the geographical location of the study region. The details of the slope ecological restoration project are shown in Table 3.

**Table 3:** The ecological restoration technology and general description of slope.

No	Technology	General description of the slope
1	Soil spray-seeding technology	The slope is located at northwest of the Ma' An Mountain, near Zha Xin road, with length of 180 m. The slope is high and steep with a slope of 70°-80°, and the highest point can reach 90 m. The total area of the slope is 10000m^2^
2	TBS galvanized wire mesh planting grass irrigation green method	The slope is located at the middle of Jiangjia Mountain with length of 600 m at bottom. The slope height is 90 m at the highest point, with a slope of 65°-80°.The total area is nearly 5000m^2^. The slope strike is NE35°, the dip is NW55°
3	High-order pellet spraying construction method	The slope is located in the middle part of Ma' An Mountain, with a length of 130–150 m at the bottom. The slope height is 50 m at the highest point, with a slope of 60°-70°. The total area of the slope is over 5500m^2^. The strike of the slope is NW45° and the dip is NE45°
4	Tape planting slope technology	The slope is located in the western section of Ma' An Mountain, with the length of 120–150 m at bottom. The slope height is 80 m at the highest point, with a slope of 60°–70°. The total area of the slope is 9000m^2^. The strike of the slope is NW35° and the dip is NE55°
5	Build the platform and pull the net green method	The slope is located in the northwest side of Ma' An Mountain, with the length of 100 m at bottom. The slope height is 80 m with a slope 65°–75°. The total area of slope is about 12000m^2^. The strike of the slope is NW30° and the dip is NE50°
6	Floating panels trenching method	The slope is located at the middle of Ma 'An Mountain, with a length of 50-80 m at the bottom. The slope height is 80 m at the highest point, with a slope of 75°–85°. The total area of the slope is 4500 m^2^. The strike of the slope is NE45° and the dip is NW45°
7	Ecological protection technology of vegetation concrete	The slope is located at the middle of Ma 'An Mountain, with a length of 100-120 m at the bottom. The slope height is 60 m at the highest point, with a slope of 65°–75°. The total area of the slope is 5000 m^2^. The strike of the slope is NW45° and the dip is NE45°

### Establishment of the evaluation index system

The selection of evaluation index of slope ecological restoration should reflect the effect of slope restoration systematically. It should not only consider the physical, mechanical and chemical characteristics of the substrate, but also consider the contribution of vegetation groups to the slope water retention and soil consolidation ability and landscape beauty. On the basis of the existing research literature^27,28^, this study combined with the actual situation and expert advice to establish a comprehensive evaluation index system from the four aspects of substrate improvement effect, ecological effect, soil and water conservation effect and landscape effect. With the help of principal component analysis, the importance of each index was compared, and finally the index system with the greatest impact on the ecological restoration effect of slope was determined. The effects of soil and water conservation includes shearing strength of root-soil composite (*X*_1_), permeability (*X*_2_), soil erosion intensity (*X*_3_), and root weight density (*X*_4_). The ecological effect includes vegetation coverage (*X*_5_), drought resistance of vegetation (*X*_6_), Shannon–Wiener diversity index (*X*_7_), Pielou evenness index (*X*_8_). Substrate improvement effect includes organic matter (*X*_9_), available *N* (*X*_10_), available *P* (*X*_11_), available *K* (*X*_12_), and soil bulk density (*X*_13_). Landscape effect includes landscape coordination (*X*_14_) and landscape capacity for visitors (*X*_15_).

According to the relevant research results, expert advice and the on-site investigation and sampling monitoring results of each slope ecological restoration sample plot, the index is divided into five grades, i.e., very good, good, normal, poor and very poor, corresponding to I, II, III, IV and V, respectively. The evaluation grade standard of the slope ecological restoration is also established, as shown in Table 4. The original data of the seven cases are shown in Table 5. Then these original data are used to prove the computing effect of the dynamic evaluation model in real complicated engineering applications.

**Table 4:** The evaluation grade standard of the slope ecological restoration.

Index	Grades				
	I	II	III	IV	V
Shearing strength of root-soil composite/kPa (*X*_1_)	(60,100]	(50,60]	(40,50]	(30,40]	(0,30]
Permeability/(mm h^−1^) (*X*_2_)	(30,40]	(20,30]	(10,20]	(5,10]	(0,5]
Soil erosion intensity/(g cm^−3^ a^−1^) (*X*_3_)	(0,5]	(5,15]	(15,25]	(25,35]	(35,45]
Root weight density/(kg m^−3^) (*X*_4_)	(3.5,4.5]	(2.5,3.5]	(1.5,2.5]	(0.5,1.5]	(0,0.5]
Vegetation coverage/% (*X*_5_)	(95,100]	(80,95]	(65,80]	(40,65]	(0,40]
Drought resistance of vegetation (*X*_6_)	1	2	3	4	5
Shannon–Wiener diversity index (*X*_7_)	(3.0,3.5]	(2.5,3.0]	(2.0,2.5]	(1.5,2.0]	(0,1.5]
Pielou evenness index (*X*_8_)	(1.0,1.2]	(0.8,1.0]	(0.6,0.8]	(0.4,0.6]	(0,0.4]
Organic matter/(g kg^−1^) (*X*_9_)	(30,40]	(20,30]	(10,20]	(5,10]	(0,5]
Available N/(mg kg^−-1^) (*X*_10_)	(75,100]	(55,75]	(35,55]	(15,35]	(0,15]
Available P/(mg kg^−1^) (*X*_11_)	(30,40]	(20,30]	(10,20]	(5,10]	(0,5]
Available K/(mg kg^−1^) (*X*_12_)	(205,260]	(150,205]	(95,150]	(40,95]	(0,40]
Soil bulk density/(g cm^−3^) (*X*_13_)	(0,1.5]	(1.5,2.0]	(2.0,2.5]	(2.5,3.0]	(3.0,3.5]
Landscape coordination (*X*_14_)	1	2	3	4	5
Landscape capacity for visitors (*X*_15_)	1	2	3	4	5

**Table 5:** The index data of the slope ecological restoration.

Index	The data of different slopes						
	1	2	3	4	5	6	7
Shearing strength of root-soil composite/kPa	39.82	50.26	54.36	58.79	35.28	49.85	75.66
Permeability/(mm h^−1^)	17.15	15.25	21.36	29.86	19.82	14.26	26.87
Soil erosion intensity/(g cm^−3^ a^−1^)	33.87	34.26	28.35	11.56	22.58	29.65	17.26
Root weight density/(kg m^−3^)	2.27	2.58	2.32	3.08	2.39	2.06	3.26
Vegetation coverage/%	30.51	86.36	90.28	97.27	35.06	48.36	95.55
Drought resistance of vegetation	5	3	2	2	5	4	2
Shannon–Wiener diversity index	1.46	2.86	2.36	2.83	1.55	1.64	2.68
Pielou evenness index	0.65	0.95	0.83	0.95	0.7	0.68	0.86
Organic matter/(g kg^−1^)	9.87	16.54	22.57	29.85	10.24	12.34	26.57
Available N/(mg kg^−1^)	38.58	50.21	58.57	68.56	35.67	45.62	63.27
Available P/(mg kg^−1^)	8.67	18.52	20.06	26.58	13.65	10.15	23.56
Available K/(mg kg^−1^)	100.25	130.25	132.52	186.52	90.42	88.67	176.53
Soil bulk density/(g cm^−3^)	2.6	2.06	2.12	1.32	2.65	2.31	1.38
Landscape coordination	4	2	2	1	3	3	1
Landscape capacity for visitors	3	2	3	2	4	3	2

### Result

**Figure 3:**
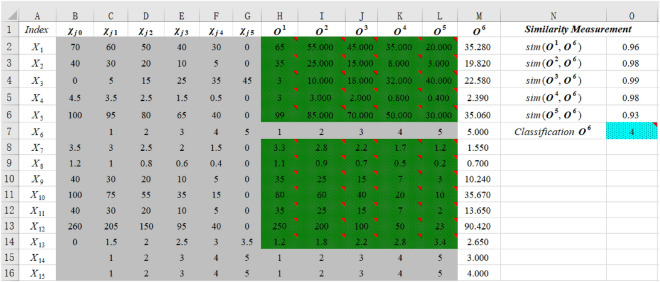
The new method for engineering slope 1 calculation implemented in an Excel spreadsheet.

According to the evaluation grade standard of the slope ecological restoration (Table 4), and the original data of the seven cases (Table 5) (Yang et al. 2019), an Excel spreadsheet is set up to perform this method, as shown in Fig. 3. Enter the Eq. (1) into the spreadsheet columns labeled *Similarity Measurement*. Enter the Eq. (2) into the spreadsheet row labeled *Classification ****Ô***. Then, the spreadsheet is loaded into the Crystal Ball software. The randomly generated evaluation objects (***O***^1^,*** O***^2^,*** O***^3^,*** O***^4^, and ***O***^5^) obey uniform distribution according to Table 2. Since the indexes *X*_6_, *X*_14_ and *X*_15_ are qualitative, the corresponding values of evaluation objects are constant, as can be seen in Fig. 3. Select the cell O7 as a predictor variable. Since, the calculated results tend to be stable when the number of trial tests exceeds 1000, it is set to 1000. Finally, the MCS is executed to calculate the probability of grade (*k*) of ***Ô***.

Based on the above the operating procedures, the evaluation results of effect of the slope ecological restoration are obtained by the proposed method. The evaluation results of the slopes are presented in Table 6. As seen in Table 6, although the results of the two methods are almost consistent and also nearly coincide with the actual situations. But the results calculated by this proposed method are more in line with the actual situation. The proposed method can obtain the evaluation results in the probabilistic form, which can quantify and reflect the variability of reconstruction effect. In addition, this method can predict the future slope ecological restoration effect and realize dynamic evaluation.

**Table 6:** Comparison evaluation results of the effect of slope ecological restoration between the two methods.

Case number	Evaluation method						The trend of the actual situation
	The proposed model					The fuzzy AHP method	
	I	II	III	IV	V		
1	0% → 62.59%	0% → 3.47%	53.12% → 12.38%	46.88% → 12.50%	0% → 9.53%	IV	The vegetation coverage was over 50% after 50d, but increased to more 90% after 1 year
2	0% → 43.9%	0% → 4.02%	99.78% → 22.95%	0.22% → 21.13%	0% → 8.98%	II	After 2 months, the vegetation coverage rate exceeded 50%, and after two years, the coverage rate exceeded 85%
3	0% → 53.2%	2.17% → 4.39%	97.83% → 7.16%	0% → 25.25%	0% → 11.11%	II	After 6 months, rock face was still exposed in a few locations. After 2 years, the vegetation coverage rate of the slope was more than 90%
4	0% → 79%	100% → 3%	0% → 3%	0% → 3%	0% → 10%	I	After 2 years, the vegetation coverage was close to 95%
5	0% → 4.71%	0.2% → 14.07%	8.27% → 3.74%	91.53% → 75.89%	0% → 1.68%	IV	After 1 year, the vegetation coverage was less than 40%
6	0% → 29.37%	0% → 7.74%	65.98% → 15.86%	34.02% → 41.41%	0% → 6.28%	III	The vegetation coverage was over 60%, but decreased to less 50% after 2 years
7	0% → 77.08%	94.52% → 3.06%	5.48% → 4.10%	0% → 5.88%	0% → 9.95%	I	After 2 years, the slope was fully covered

For example, the vegetation coverage of the slope 1 was over 50% after 50d, but increased to more 90% after 1 year. The evaluation result obtained by the fuzzy AHP method is class IV, which cannot reflect the variability of the reconstruction effect. According to the results obtained by this proposed model, it can be clearly found that the initial evaluation result is grade III with a probability of 53.12%, and the future evaluation result changes to grade I with a probability of 62.5%, which can reflect the variability of the restoration effect. We can also find that the calculation results of slopes 2 and 6 are in good agreement with the actual situation, but the traditional method cannot obtain the variability of the result. The evaluation results of slope 4 and 7 obtained by the fuzzy AHP method are class I. However, compared with the actual situation, the vegetation coverage rate of slope 4 did not reach more than 95% until two years later. The vegetation of slope 7 completely covered the rocky slope after 50 days, and after 2 years it basically formed a natural landscape in harmony with the surrounding environment. The results obtained by this model are closer to the reality. The initial evaluation result of slope 4 is grade II, with a probability of 100%. Over time, the evaluation result changes to grade I, with a probability of 79%. The initial evaluation result of slope 7 was grade II, with a probability of 94.52%. With the passage of time, the evaluation grade result changes to grade I, with a probability of 77.08%.

Consequently, it can be concluded that the robustness of the proposed method surpasses the robustness of traditional methods, such as the entropy weight method, and its application is also more convenient than that of traditional methods, which makes it a suitable method for assessing the effect of slope ecological restoration. Likewise, the proposed method can obtain the evaluation results in the probabilistic form, which can quantify and reflect the variability of reconstruction effect. However, due to need determine the weights of indexes, the computing efficiency of the entropy weight method is poor. In order to solve the problem of numerous indexes and tremendous data, the fuzzy AHP method is not very satisfactory, resulting very specialized and time consuming.

### Discussion

Due to the spatial effect of the site and the influence of the experimental instruments, some indexes in the same site are different, but might follow a certain distribution. Therefore, it is necessary to consider the uncertainty of the index and analyze the index sensitivity in order to illustrate the performance of the proposed method. In a mathematical sense, index sensitivity can be understood as the degree of change in function *F*(*x*) caused by a small change in variable *x*. Since the global sensitivity analysis requires multiple calls to the simulation model, which takes a long time, this paper uses the local sensitivity analysis method to analyze the sensitivity of the index *S*.

9 $$S = \left| {\frac{{\frac{{F\left( {x_{1} , \ldots ,x_{i} + \Delta x_{i} , \ldots ,x_{n} } \right) - F\left( {x_{1} , \ldots ,x_{i} , \ldots ,x_{n} } \right)}}{{F\left( {x_{1} , \ldots ,x_{i} , \ldots ,x_{n} } \right)}}}}{{\frac{{\Delta x_{i} }}{{x_{i} }}}}} \right|$$

The indexes of the investigated object (***Ô***) are independent of each other and assumed to be uncertain with normal distribution. Based on the data of these 7 slope ecological restoration methods, it is assumed that all index data obey normal distribution, the mean value is the original data of each index, and the standard deviation is 2. Then the sensitivity difference of each index was calculated by using this proposed model and the fuzzy AHP method.

**Figure 4:**
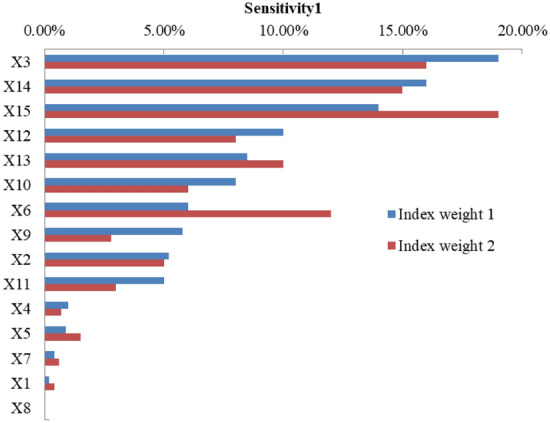
Indexes sensitivity of solpe1 using the fuzzy AHP method.

It can be seen from Fig. 4 that when fuzzy AHP is used to calculate the index sensitivity, the sensitivity of indexes are obvious differences for two different indexes weights, which can also be said that the calculation results are greatly influenced by individual subjective feelings. However, the proposed method does not consider the influence of index weight, resulting in relatively stable results, so the calculation performance has greater advantages compared with fuzzy AHP.

**Figure 5:**
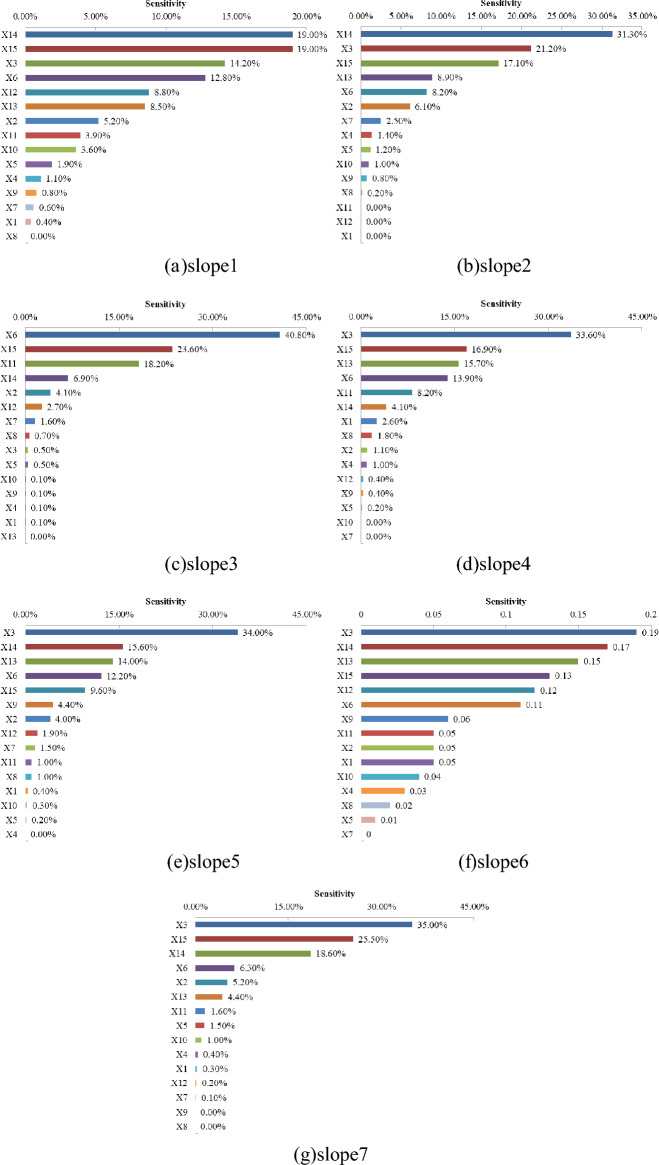
Sensitivity of indexes under different ecological restoration methods.

It can be seen from Fig. 5 that for the first method (soil spray-seeding technology), the indexes with the highest sensitivity are *X*_14_, *X*_15_ and *X*_3_, which can also be proved in practice that the scientific and reasonable allocation of grass irrigation species, the durability and erosion resistance of the substrate have the greatest influence on the restoration effect. In the second method, the indexes with greater sensitivity are *X*_14_, *X*_3_ and *X*_15_, which are basically consistent with the sensitivity results of the first method. Also, in practice, whether the barbed wire is sturdy and durable directly affects the stability of the ecological substrate on the slope. The indexes with high sensitivity of the third method are *X*_6_, *X*_15_ and *X*_11_, which are significantly different from the results of the first and second methods. The drought resistance of vegetation and available *P* has a great impact on the ecological restoration effect. In engineering practice, the most critical method is to use plant fibers to enhance the soil and water conservation capacity of the substrate and require the use of surface guest soil rich in organic matter and clay particles. The index sensitivity degree of the fourth to seventh methods has a small difference, and the index with the largest sensitivity degree is *X*_3_, which is basically consistent with the results in engineering practice.

Different grading standards of the effect of slope ecological restoration may lead to changes in evaluation results obtained using this proposed method. Because the similarity results *sim*(***O***^*i*^, ***Ô***)(*i* = 1, 2, 3, 4, 5) are directly related to the objects (***O***^1^,*** O***^2^,*** O***^3^,*** O***^4^, and ***O***^5^) randomly generated from the evaluation grade standard of the slope ecological restoration. Hence, it can be known that completely avoiding subjective influence during the evaluation is impossible. In order to improve the calculation efficiency and reduce subjectivity influence, it is the focus of our next study to classify the effect of the slope ecological restoration without the evaluation grade standard. In brief, by comprehensively using the CS and MC to quantitatively estimate the dynamic effect of the slope ecological restoration, it can accurately provide scientific basis for developing the slope ecological restoration measures.

## Conclusions

Herein an evaluation method based on CS and MC was proposed to dynamically evaluate the effect of slope ecological restoration. The proposed method has the advantages of avoiding the influence of subjectivity in indexes weights and obtaining the dynamic evaluation results in the probabilistic form. Meanwhile, the proposed evaluation method can take into account the uncertainty of the indexes of the ecological restoration of slopes. Taking several typical engineering slopes as examples, the effectiveness of the proposed method is verified by dynamic evaluation results and sensitivity degree of indexes. The results obtained by the proposed method are more consistent with the actual situations than the traditional methods. Due to the lack of a large number of slope ecological restoration data, the method of determining the transformation probability matrix in this study is still insufficient. Subsequent research will improve the determination method of the transfer probability matrix by combining a large number of slope restoration data.

## Data Availability

All data generated or analyzed during this study were included in this article.
